# Statistical Analysis of the Exchange Rate of Bitcoin

**DOI:** 10.1371/journal.pone.0133678

**Published:** 2015-07-29

**Authors:** Jeffrey Chu, Saralees Nadarajah, Stephen Chan

**Affiliations:** School of Mathematics, University of Manchester, Manchester M13 9PL, United Kingdom; Max Planck Institute for the Physics of Complex Systems, GERMANY

## Abstract

Bitcoin, the first electronic payment system, is becoming a popular currency. We provide a statistical analysis of the log-returns of the exchange rate of Bitcoin versus the United States Dollar. Fifteen of the most popular parametric distributions in finance are fitted to the log-returns. The generalized hyperbolic distribution is shown to give the best fit. Predictions are given for future values of the exchange rate.

## Introduction

Introduced and first documented by Satoshi Nakamoto in 2009, Bitcoin is a form of cryptocurrency—an “electronic payment system based on cryptographic proof” [1], instead of traditional trust. [1] noted that buying and selling online has become reliant “almost exclusively on financial institutions serving as trusted third parties to process electronic payments”. In other words, payments for online transactions must go through a company, such as a bank or credit card issuer, to be checked for factors such as fraud and successful payment. This kind of system is based on trust, however these checks come at a price in the form of increased transaction costs [1], meaning that we often see restrictions in the form of minimum spend limits for electronic payments—i.e., on credit or debit cards. Bitcoin transactions are non-reversible—they are “computationally impractical to reverse” [1] and can help to reduce fraud.

Interest in Bitcoin has grown at an increasing pace in recent years. At the end of August 2013, the total available Bitcoins were valued at over 1.5 billion United Stated Dollars (USDs), and in December 2013 the processing power of the Bitcoin network was claimed to be “roughly 300 times the combined power of the top 500 supercomputers” [2]. [3] states that this is because supporters of Bitcoin see it as “an ideal currency for mainstream consumers and merchants”. In short, the high liquidity, reduced costs and the high speed of Bitcoin’s partially anonymous system are what make this currency so interesting [3].

From a wider perspective, Bitcoin is not currently controlled by a central governing body, reducing privacy concerns. In addition, Bitcoin is not linked with any type of commodity, for example, gold or silver [4]. Due to the decentralised nature of Bitcoin, the network is instead controlled by its users. The Bitcoin system utilises a peer-to-peer network of all those who are involved in creating and trading Bitcoins, to process and check all transactions. Therefore “each participant is obliged to maintain the entire transaction history of the system rendering all transactions transparent” [4]. This in theory should create an incentive for all users to protect the Bitcoin network. The freedom of Bitcoin may also allow organisations such as WikiLeaks to be funded and to carry out business with fewer restrictions. However, this freedom along with increased interest and adoption from users means that it may aid and “facilitate money laundering, tax evasion and trade in illegal drugs and child pornography” [3].

Bitcoin has properties which could make it important in commerce, the most significant being low transaction costs [5]. As there is essentially no middle man when performing transactions using Bitcoins, “there are few, if any, transaction fees associated with transfers” [6]. This is in comparison to traditional payment methods which can have significantly higher transaction fees. Thus, in some cases, Bitcoin could provide a more feasible alternative payment method [6]. This has implications in the developed world, for example, allowing individuals and businesses to carry out online transactions with little or no fees, reducing overall costs. In particular, for transactions which require conversions between different currencies (often incurring exchange rate fees), Bitcoin could offer a simpler and more universal payment system.

Similarly, for less economically developed countries (for simple monetary transfers between two parties) services such as Western Union have traditionally been a popular way to send money back home from overseas, or to another party within the same country. A flat or percentage fee is often incurred whilst sending money. Again, Bitcoin could allow for money to be quickly and securely transferred, without the need for any additional fees. This would be hugely beneficial to those from less economically developed countries.

Traditional purchase of goods and services online is dominated by credit and debit cards, or PayPal. But where other digital currencies have failed to get a foothold, Bitcoin may not necessarily succeed. [3] suggests that even if card use is becoming less popular, companies may be able to reduce transaction fees in general, to compete with Bitcoin. On the other hand, Bitcoin may instead be able to establish itself as a standard in micropayments. The relative cost of processing lower value transactions is much greater for traditional payment methods, thus Bitcoin has a competitive advantage [3].

Bitcoin as an international payment standard has its benefits, but its volatile price suggests that it may still suffer from problems of traditional currencies. Therefore, Bitcoin could be considered as a currency exchange rate. However, some researchers argue that Bitcoin does not fulfil the criteria for it to be considered as a true currency. [4] claim that “Bitcoin is not a denominated fiat currency”, however it has features similar to cash, for example, irreversibility and partial anonymity. According to [7], the wild fluctuations in Bitcoin price cannot be explained by economic and financial theory. Factors such as interest rates and inflation do not exist, as there is no central bank overseeing the issuing of Bitcoin. Thus, Bitcoin price is “driven solely by the investors’ faith in the perpetual growth” [7]. Further to this, [5] indicates that the three criteria for Bitcoin to be a currency, being a unit of account; medium of exchange; store of value; are not sufficiently met. International use of Bitcoin is still very limited, “indicating that few people use it widely as a medium of exchange” [5]; Bitcoin can be traded on various exchanges usually at different prices; the daily exchange against USD shows little correlation with USD exchange rate against other major currencies.

Although Bitcoin can be considered to be relatively new, there has already been some initial analysis into the cryptocurrency, and we provide a literature review here.

[2] study the links between social signals and Bitcoin price through a social feedback cycle. Using data from Bitcoin exchanges, social media, Google search trends and the user base of Bitcoin, they found two main positive feedback loops, social and user adoption cycle. An increase in popularity of Bitcoin leads to increases in searches for Bitcoin and more social media coverage. Increases in the number of users leads to an increase in Bitcoin popularity and coverage which contributes to the effect of the social cycle. However, their results fail in explaining sudden negative changes in Bitcoin price.

[7] studies the relationship between digital currencies, such as Bitcoin, and search queries through Google Trends and Wikipedia. Price level was shown to be significantly positively related to search terms, with the relation being bi-directional, in that searches affects prices and prices affect searches.

[8] provide an empirical analysis of Bitcoin-Exchange Risk. They note that whilst Bitcoin has seen the greatest adoption of any cryptocurrency thus far, it has also attracted the attention of criminals. Focusing on the risk of Bitcoin users from currency exchanges, their survival analysis shows that “exchange probability of closure is inversely correlated to its trade volumes” [8]. Supporting this analysis, there is an indication that “popular exchanges are more likely to suffer security breaches” [8], something which one might expect.

[9]’s analysis looked into whether Bitcoin intra-network transaction and on-exchange trading volumes are linked, and also tries to determine if Bitcoin can be classed as an asset or a currency. Using data from 2011 to 2013, including trading data, transaction data and important Bitcoin dates, results indicate that the interest generated from new users of Bitcoin impacts on the volume of Bitcoins traded at the Bitcoin exchange, but not in the overall system. The authors note that as a currency, Bitcoin would need to be a “means of trade, a vehicle to store value, or a unit of account in order to compare the value of different goods or services” [9]. Thereby hypothesising that increased adoption of Bitcoin will increase overall Bitcoin network volume. However, if Bitcoin is an asset, the hypothesis is that an increase in Bitcoin adoption is positively linked to an increase in Bitcoin exchange volume. Therefore, from the results it appears that new users adopt Bitcoin with “speculative investment” as an objective, rather than using it as currency to purchase goods and services.

Some of the latest research comes from [10], modelling and predicting the Bitcoin/USD exchange rate through the application of a non-causal autoregressive model. Using data from daily closing rates of Bitcoin/USD from February 2013-June 2013, results from the analysis show that the Bitcoin/USD rate “displays episodes of local trends, which can be modelled and interpreted as speculative bubbles” [10]. [10] suggest that these speculative bubbles may arise as a result of speculative trading of Bitcoin—further supporting [9]’s conclusion that new Bitcoin users treat it as an asset.

[11] look at the structure and evolution of the Bitcoin transaction network. The study shows two phases in the lifetime of the Bitcoin system, initially when user adoption was low, Bitcoin was “more of an experiment than a real currency”. However, after it started to gain momentum, Bitcoin started to behave more like a real currency. In addition, they found that in the second phase the accumulation of Bitcoins through wealth distribution converges to a stable stretched exponential distribution.

The study of [4] measures volatility of Bitcoin exchange rate against six major currencies. Using raw annualised data over a four year period from 2010 to 2014 and adjusted data, taking account of volume of transactions, they find that Bitcoin shows the highest annualised volatility of percentage change in daily exchange rates. However, accounting for the (low) volume of Bitcoin trades, volatility of the Bitcoin exchange rate is significantly reduced, showing a more stable exchange rate. The authors note that claims of volatility and risk in Bitcoin should be interpreted carefully. The significance of the low trading volume of Bitcoin means that the volatility of Bitcoin will appear greater, and any trading will have a greater effect than with traditional currency.

Using the data for the period 2010–2013, [12] show “Bitcoin investment exhibits very high volatility but also very high returns. In addition, for holders of well diversified portfolios, high risk is compensated by low correlations with other assets. Including even a small proportion of Bitcoins in a well-diversified portfolio may dramatically improve risk-return characteristics”.

Using a known technique that is robust in detecting bubbles, [13] investigated the existence of bubbles in the Bitcoin market. They detected a number of short-lived bubbles over the period 2010–2014. Three of these were huge appearing in the latter part of the period 2011–2013 and lasting from 66 to 106 days.

Through wavelet coherence analysis, [14] examines Bitcoin price formation and the main drivers of price. The study shows that factors such as “use in trade, money supply and price level” have an impact on long term price. A general increase in price attracts people to create Bitcoins, thus profit arises from the creation of Bitcoins over time. Although price is determined through supply and demand, it is also influenced by the interest of investors. In periods of significant growth or decline in price, good and bad news were found to push the price further up or down, respectively.

As there is no intermediary, there is no bid-ask spread for the Bitcoin exchange rate. The lack of bid-ask spreads, that is, the absence of transaction costs, can effect the movement of quote prices, hence shape the statistical properties or returns. There is a huge literature on the effects of transaction costs and bid-ask spreads on returns: [15] find evidence to suggest that “market-observed average returns are an increasing function of the spread; asset returns to their holders, net of trading costs, increase with the spread; and, there is a clientele effect, whereby stocks with higher spreads are held by investors with longer holding periods”; [16] find evidence to suggest that “returns on high-spreads stocks are higher, but less spread-sensitive, than the returns on low-spread stocks”; [17] finds evidence to suggest that serial covariances of returns are strongly negatively correlated with the square of quoted spreads; [18] find evidence to suggest that quoted spreads are larger when larger trades take place; and so on. This suggests that any effect on the return of Bitcoin must be related to other factors such as news relating to the digital currency.

Two recent papers on fitting of distributions to exchange rate data (not just Bitcoin) are [19] and [20]. [19] fitted the generalized Lambda, skew *t*, normal inverse Gaussian and normal distributions as well as the Johnson’s family of distributions to the data. [20] fitted the Student’s *t*, asymmetric Student’s *t*, hyperbolic, generalized hyperbolic, generalized Lambda, skew *t*, normal inverse Gaussian and normal distributions to the data.

One of the known features of Bitcoin is that it is highly volatile, see, for example, [4] and [12]. Hence, accurate fitting of its variation is so important. The aim of this paper is to provide a formal statistical analysis of the exchange rate of Bitcoin versus the USD using a wide range of known parametric distributions in finance. The statistical analysis presented is the most comprehensive using parametric distributions for any kind of exchange rate data.

Other motivation for this paper are: i) the exchange rate of Bitcoin to the USD behaves very differently to the exchange rate of major currencies, see Section 2; ii) there have been studies investigating the best fitting distributions for the exchange rate of major currencies, but none so far for the exchange rate of Bitcoin; iii) risk measures like the value at risk and expected shortfall can be easily computed from the fitted parametric distributions; iv) out of sample values can be easily predicted from the fitted parametric distributions.

The contents of this paper are organized as follows. Section 2 presents the Bitcoin data used here. Some summary features of the data are described. Section 3 presents fifteen of the most popular parametric distributions in finance. Several of these distributions were introduced in the last few years. Section 4 analyzes the exchange rate data for Bitcoin using the distributions in Section 3. Among other things future predictions are given for the exchange rate. Finally, some conclusions and future work are noted in Section 5.

## Data

The data are daily Bitcoin Exchange Rate on Bitstamp (Bitcoin versus USD) from the 13th of September 2011 to the 8th of May 2014. The data were obtained from the database Quandl, see https://www.quandl.com/data/BITCOIN/BITSTAMPUSD Note that there are no data before the 13th of September 2011. We have chosen to use data from the Bitstamp Bitcoin exchange instead of the Bitcoin Price Index published by CoinDesk for the following reasons. We wanted to focus on a specific Bitcoin exchange based in Britain, and one which has a significant trading volume. Bitstamp fulfils both these criteria, being located in London and being the world’s second largest Bitcoin trading volume. Bitstamp exchange started trading on 13th September 2011, however, CoinDesk launched its Bitcoin Price Index only on 11th September 2013. Therefore, we feel that using the Bitcoin Price Index would lead to a sample size which may be too small and unreliable to conclude any results from. The Bitcoin Price Index represents an average of Bitcoin prices across leading global exchanges. Therefore, the index is easily affected when a certain exchange approaches a downturn or a suspension. In addition, the Bitcoin Price Index omits Bitcoin exchange if the price is not updated for more than thirty minutes. Overall, both the Bitcoin Index Price and Bitstamp exchange price overlap and follow each other very closely, so there is little difference from choosing one over the other.

**Fig 1:**
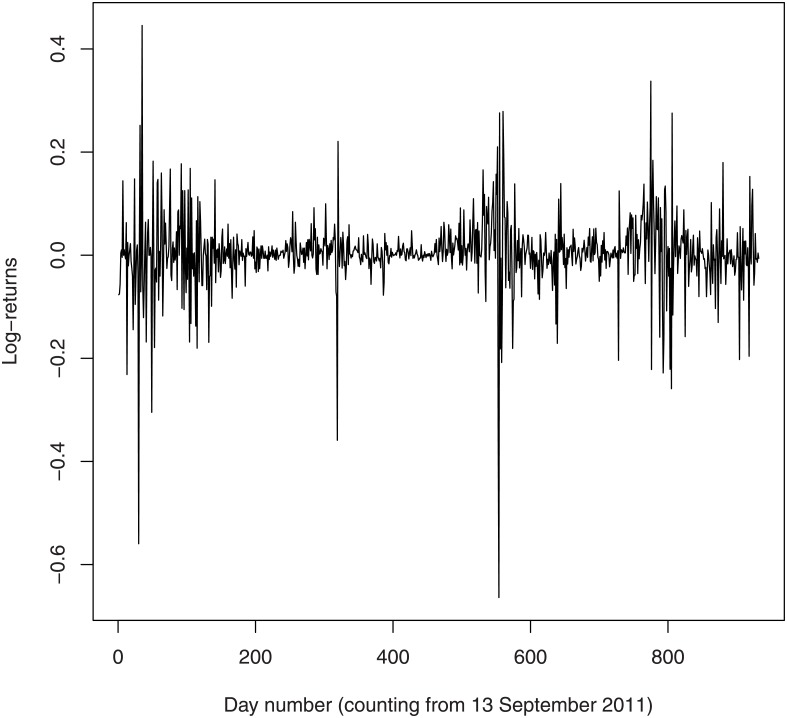
Log-returns of the exchange rate of Bitcoin.

The log-returns of the exchange rate are plotted in Fig 1. Some summary statistics of the log-returns are given in Table 1. We see that the log-returns have mean and median almost equal to zero, are negatively skewed and have a peakedness greater than that of the normal distribution. Also shown in Table 1 are the summary statistics of the exchange rates to USD of some of the major currencies: Australian Dollar, Brazilian Real, Canadian Dollar, Swiss Franc, Euro, British Pound and Japanese Yen. We see that the behavior of Bitcoin is sharply different compared to these currencies: its minimum is much smaller, its first quartile is much smaller, its median is much larger, its mean is much larger, its third quartile is much larger, its maximum is much larger, its interquartile range is much wider, its range is much wider, its skewness is much more negative, its kurtosis is much more peaked, its standard deviation is much larger, its variance is much larger and its coefficient of variation is much smaller. These results are consistent with findings in [4] and [5].

**Table 1:** Summary statistics log-returns of the exchange rate of Bitcoin versus those of Australian Dollar, Brazilian Real, Canadian Dollar, Swiss Franc, Euro, British Pound and Japanese Yen.

Statistics	Bitcoin	OZ	BR	CA	CH	EU	UK	JP
Minimum	−0.664	-0.067	-0.118	-0.050	-0.055	-0.046	-0.045	-0.046
First quartile	−0.012	-0.004	-0.005	-0.003	-0.004	-0.004	-0.003	-0.003
Median	0.004	-0.0002	-0.00005	0	0	0	-0.00006	0.00009
Mean	0.005	-0.00005	0.0001	-0.00005	-0.0001	-0.00004	0.00001	0.00004
Third quartile	0.025	0.004	0.004	0.003	0.004	0.003	0.003	0.004
Maximum	0.446	0.088	0.097	0.043	0.085	0.038	0.039	0.037
Interquartile range	0.037	0.008	0.009	0.006	0.008	0.007	0.006	0.007
Range	1.109	0.155	0.215	0.094	0.139	0.085	0.084	0.083
Skewness	−1.503	0.866	0.110	-0.076	0.358	-0.145	0.055	-0.253
Kurtosis	22.425	12.707	13.826	5.765	9.170	2.662	4.413	3.889
Standard deviation	0.069	0.008	0.010	0.006	0.007	0.006	0.006	0.006
Variance	0.005	0.00007	0.0001	0.00003	0.00005	0.00004	0.00003	0.00004
Coefficient of variation	15.156	-157.291	96.649	-108.894	-57.625	-143.498	419.938	147.998

Fitting of a statistical distribution usually assumes that the data are independent and identically distributed (i.e., randomness), have no serial correlation, and have no heteroskedasticity.

**Fig 2:**
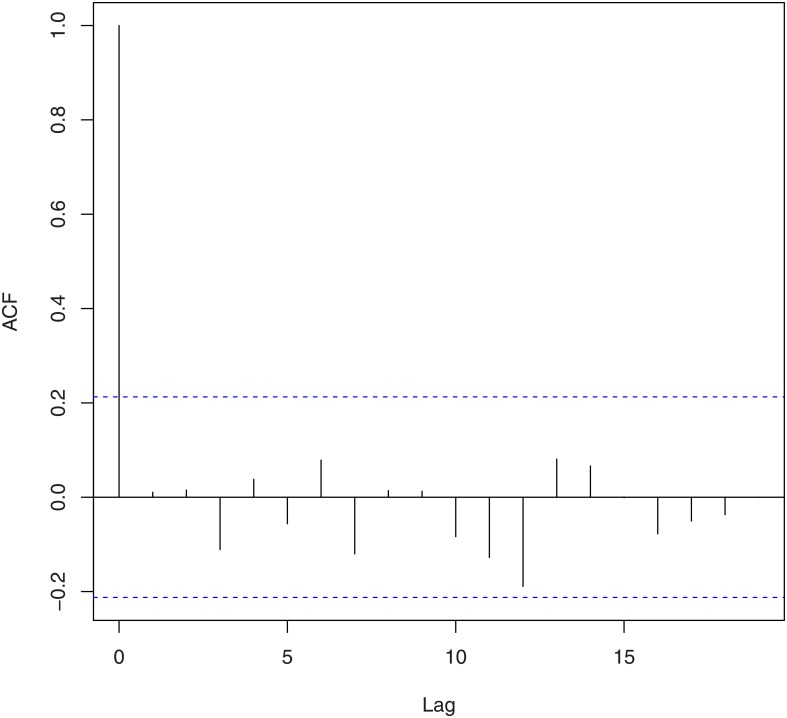
Autocorrelation function of the log-returns of the exchange rate of Bitcoin.

**Fig 3:**
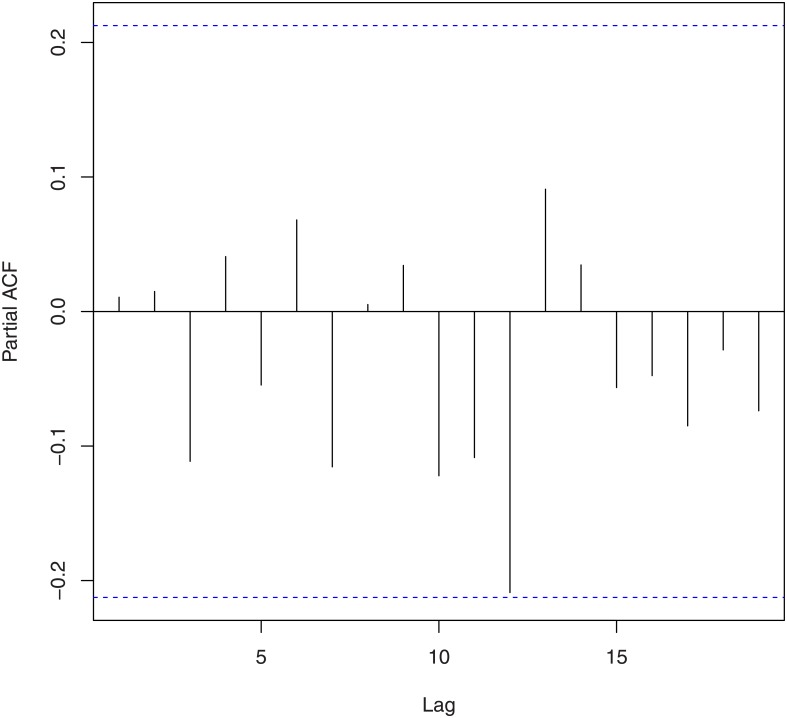
Partial autocorrelation function of the log-returns of the exchange rate of Bitcoin.

**Fig 4:**
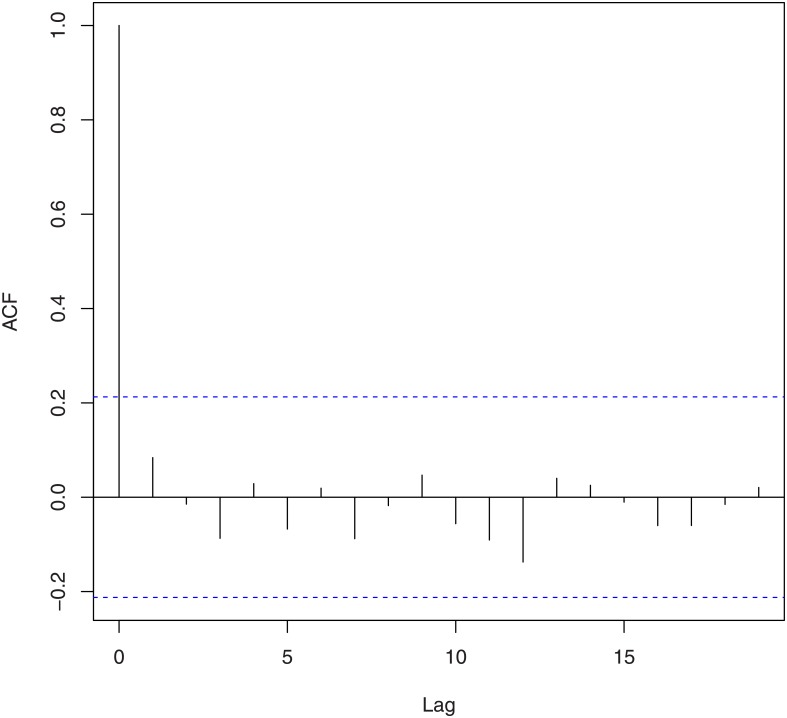
Autocorrelation function of the squared log-returns of the exchange rate of Bitcoin.

**Fig 5:**
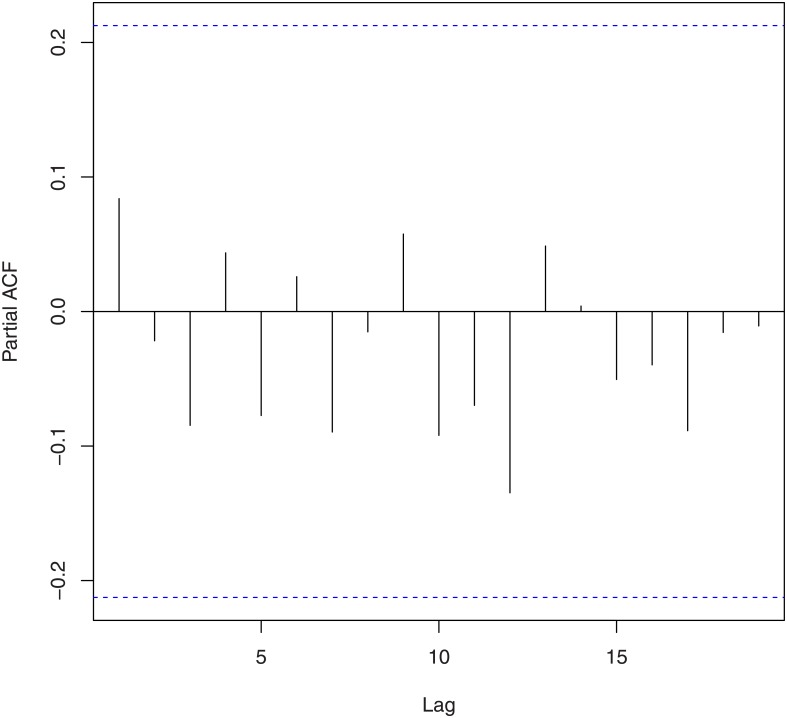
Partial autocorrelation function of the squared log-returns of the exchange rate of Bitcoin.

We tested for randomness using [21]’s rank test, [22]’s test, the difference sign test, the rank test, [23]’s runs test, the turning point test, and the test due to [24] and [25]. The corresponding *p*-values based on log-returns and squares of log-returns are given in Table 2. We tested for no serial correlation using [26] [27] [28]’s method and the method due to [29] and [30]. The corresponding *p*-values based on log-returns and squares of log-returns are given in Table 3. These values are supported by the plots of the autocorrelation function and the partial autocorrelation function shown in Figs 2, 3, 4 and 5. We tested for no heteroskedasticity using [31]’s test. The corresponding *p*-values based on log-returns and squares of log-returns are given in Table 4. All of the tests performed in Tables 2, 3 and 4 are non-parametric in nature, i.e., no distributional assumptions are made about the data.

**Table 2:** *p*-values of the tests for randomness.

Test	*p*-values using	
	log-returns	log-returns^2^
Bartels (1982)	0.123	0.066
Cox and Stuart (1955)	0.613	0.433
Difference sign	0.238	0.112
Rank	0.352	0.223
Wald and Wolfowitz (1940)	0.243	0.202
Turning point	0.129	0.121
Box and Pierce (1970)	0.302	0.256

**Table 3:** *p*-values of the tests for no serial correlation.

Test	*p*-values using	
	log-returns	log-returns^2^
Durbin and Watson	0.839	0.766
Godfrey and Breusch	0.297	0.265

**Table 4:** *p*-values of the tests for no heteroskedasticity.

Test	*p*-values using	
	log-returns	log-returns^2^
Breusch and Pagan	0.403	0.299

It is not uncommon that exchange rate data are independent and identically distributed, have no serial correlation, and have no heteroskedasticity. Some published examples of such exchange rate data can be found in [19] and [20].

Section 3 discusses fifteen distributions for the log-returns of the exchange rate of Bitcoin. These distributions are the normal, Student’ *t*, logistic, Laplace, exponential power, skew normal, skew *t*, generalized *t*, skewed exponential power, asymmetric exponential power, skewed Student’s *t*, asymmetric Student’s *t*, normal inverse gamma, hyperbolic and generalized hyperbolic distributions. The choice of these fifteen distributions is not arbitrary. Some of these distributions have been used by many others to fit exchange rate data: [32] fitted the normal distribution to the exchange rates of Japanese Yen to USD, Deutsche Mark to USD, British Pound to USD, Japanese Yen to Deutsche Mark, Japanese Yen to British Pound and Deutsche Mark to British Pound; [33] fitted the hyperbolic distribution; [34] fitted the skewed *t* distribution to the exchange rate of Polish Zloty to USD; [35] fitted the Laplace distribution to the exchange rate of Euro to USD; [36] fitted the Student’s *t* and skewed Student’s *t* distributions to the exchange rates of British Pound to USD, Euro to USD and Japanese Yen to USD; [37] fitted the skewed *t* distribution to the exchange rates of Mexican Peso, Brazilian Real, Euro, Swiss Franc, Canadian Dollar, Japanese Yen, British Pound and Australian Dollar to USD; [38] fitted the normal, Student’s *t* and normal inverse gamma distributions to the exchange rates of British Pound, Canadian Dollar, Euro, Deutsche Mark, Japanese Yen, and Swiss Franc to USD; [39] fitted the normal distribution to the exchange rate of Peso in Mexico to USD; [40] fitted the skewed Student’s *t* and generalized hyperbolic distributions to the exchange rates of Euro and Japanese Yen to USD; [19] fitted the skew *t*, normal inverse Gaussian and normal distributions to the exchange rates of Japanese Yen, Brazilian Real, Australian Dollar, Canadian Dollar, Swiss Franc, Euro, British Pound, Mexican Peso and Turkish Lira to USD; [20] fitted the skew *t*, normal inverse Gaussian, normal, Student’s *t*, asymmetric Student’s *t*, hyperbolic and generalized hyperbolic distributions to the same exchange rate data as in [19]; and so on. We feel that the collection used here is the most comprehensive collection of distributions used to analyze any exchange rate data set anywhere.

## Distributions Fitted

Let *X* denote a continuous random variable representing the log-returns of the exchange rate of Bitcoin. Let *f*(*x*) denote the probability density function (pdf) of *X*. Let *F*(*x*) denote the cumulative distribution function (cdf) of *X*. We suppose *X* follows one of fifteen possible distributions, the most popular parametric distributions used in finance. They are specified as follows:

- the normal distribution [41], [42] with
  $$f\left(x\right)=\frac{1}{\sqrt{2\pi }\sigma }\exp\;\{-\frac{{\left(x-\mu \right)}^{2}}{2\sigma^{2}}\}$$
  for −∞ < *x* < ∞, −∞ < *μ* < ∞ and *σ* > 0;
- the Student’s *t* distribution [43] with
  $$f\left(x\right)=\frac{K\left(\nu \right)}{\sigma }{\left[1+\frac{{\left(x-\mu \right)}^{2}}{\sigma^{2}\nu }\right]}^{-\left(1+\nu \right)/2}$$
  for −∞ < *x* < ∞, −∞ < *μ* < ∞, *σ* > 0 and *ν* > 0, where $K(\nu )=\sqrt{\nu }B\left(\nu /2,1/2\right)$ and *B*(⋅, ⋅) denotes the beta function defined by
  $$B\left(a,b\right)=\int_{0}^{1}t^{a-1}{\left(1-t\right)}^{b-1}dt;$$
- the logistic distribution with
  $$f\left(x\right)=\frac{1}{\sigma }\text{exp}\left(-\frac{x-\mu }{\sigma }\right){\left\{1+\text{exp}\left(-\frac{x-\mu }{\sigma }\right)\right\}}^{-2}$$
  for −∞ < *x* < ∞, −∞ < *μ* < ∞ and *σ* > 0;
- the Laplace distribution [44] with
  $$f\left(x\right)=\frac{1}{2\sigma }\exp\;(-\frac{|x-\mu |}{\sigma })$$
  for −∞ < *x* < ∞, −∞ < *μ* < ∞ and *σ* > 0;
- the exponential power distribution [45] with
  $$f\left(x\right)=\frac{\beta }{2\sigma \Gamma \left(1/\beta \right)}\text{exp}\left\{-{\left(\frac{\left|x-\mu \right|}{\sigma }\right)}^{\beta }\right\}$$
  for −∞ < *x* < ∞, −∞ < *μ* < ∞, *σ* > 0 and *β* > 0, where Γ(⋅) denotes the gamma function defined by
  $$\Gamma \left(a\right)=\int_{0}^{\infty }t^{a-1}\exp\;\left(-t\right)dt;$$
- the skew normal distribution [46] with
  $$f\left(x\right)=\frac{2}{\sigma }\phi (\frac{x-\mu }{\sigma })\Phi (\lambda \frac{x-\mu }{\sigma })$$
  for −∞ < *x* < ∞, −∞ < *μ* < ∞, −∞ < λ < ∞ and *σ* > 0, where *ϕ*(⋅) and Φ(⋅) denote, respectively, the pdf and the cdf of the standard normal distribution;
- the skew *t* distribution [47] with
  $$\begin{array}{ll} f\left(x\right)= & \frac{K\left(\nu \right)}{\sigma }{\left[1+\frac{{\left(x-\mu \right)}^{2}}{\sigma^{2}\nu }\right]}^{-\left(1+\nu \right)/2}\\ & +\frac{2K^{2}\left(\nu \right)\lambda \left(x-\mu \right)}{\sigma^{2}}{\;}_{2}F_{1}\left(\frac{1}{2},\frac{1+\nu }{2};\frac{3}{2};-\frac{\lambda^{2}{\left(x-\mu \right)}^{2}}{\sigma^{2}\nu }\right) \end{array}$$
  for −∞ < *x* < ∞, −∞ < *μ* < ∞, −∞ < *λ* < ∞, *σ* > 0 and *ν* > 0, where _2_ *F*_1_(*a*, *b*; *c*; *x*) denotes the Gauss hypergeometric function defined by
  $${}_{2}F_{1}\left(a,b;c;x\right)=\overset{\infty }{\underset{k=0}{\sum }}\frac{{\left(a\right)}_{k}{\left(b\right)}_{k}}{{\left(c\right)}_{k}}\frac{x^{k}}{k!},$$
  where (*e*)_*k*_ = *e*(*e* + 1)⋯(*e* + *k* − 1) denotes the ascending factorial;
- the generalized *t* distribution [48] with
  $$f\left(x\right)=\frac{\tau }{2\sigma \nu^{1/\nu }B\left(\nu ,1/\tau \right)}{\;\left[1+\frac{1}{\nu }{\left|\frac{x-\mu }{\sigma }\right|}^{\tau }\right]}^{-\left(\nu +1/\tau \right)}$$
  for −∞ < *x* < ∞, −∞ < *μ* < ∞, *σ* > 0, *ν* > 0 and *τ* > 0;
- the skewed exponential power distribution [49] with
  $$f\left(x\right)=C\{\begin{array}{ll} \exp\;\left\{-\frac{1}{p}{\left[\frac{\mu -x}{2\sigma \alpha }\right]}^{p}\right\}, & \text{if}\;x\leq \mu \text{,}\\ \exp\;\left\{-\frac{1}{p}{\left[\frac{x-\mu }{2\sigma \left(1-\alpha \right)}\right]}^{p}\right\}, & \text{if}\;x>\mu \end{array}$$
  for −∞ < *x* < ∞, −∞ < *μ* < ∞, *α* > 0, *σ* > 0 and *p* > 0, where *C* = 1/[2*σA*_0_ (*p*)] and *A*_0_(*x*) = *x* ^(1/*x*)−1^Γ(1/*x*);
- the asymmetric exponential power distribution [49] with
  $$f\left(x\right)=C\{\begin{array}{ll} \exp\;\left\{-\frac{1}{p_{1}}{\left[\frac{\mu -x}{2\sigma \alpha }\right]}^{p_{1}}\right\}, & \text{if}\;x\leq \mu \text{,}\\ \exp\;\left\{-\frac{1}{p_{2}}{\left[\frac{x-\mu }{2\sigma \left(1-\alpha \right)}\right]}^{p_{2}}\right\}, & \text{if}\;x>\mu \end{array}$$
  for −∞ < *x* < ∞, −∞ < *μ* < ∞, *σ* > 0, *α* > 0, *p*_1_ > 0 and *p*_2_ > 0, where *C* is given by
  $$C=\frac{1}{2\sigma \alpha A_{0}(p_{1})+2\sigma \left(1-\alpha \right)A_{0}(p_{2})};$$
- the skewed Student’s *t* distribution [50] with
  $$f\left(x\right)=\frac{K\left(\nu \right)}{\sigma }\{\begin{array}{ll} {\left\{1+\frac{1}{\nu }{\left[\frac{x-\mu }{2\sigma \alpha }\right]}^{2}\right\}}^{-\frac{\nu +1}{2}}, & \text{if}\;x\leq \mu \text{,}\\ {\left\{1+\frac{1}{\nu }{\left[\frac{x-\mu }{2\sigma \left(1-\alpha \right)}\right]}^{2}\right\}}^{-\frac{\nu +1}{2}}, & \text{if}\;x>\mu \end{array}$$
  for −∞ < *x* < ∞, −∞ < *μ* < ∞, 0 < *α* < 1 and *ν* > 0;
- the asymmetric Student’s *t* distribution [50] with
  $$f\left(x\right)=\frac{1}{\sigma }\{\begin{array}{ll} \frac{\alpha }{\alpha^{*}}K\left(\nu_{1}\right){\left\{1+\frac{1}{\nu_{1}}{\left[\frac{x-\mu }{2\sigma \alpha^{*}}\right]}^{2}\right\}}^{-\frac{\nu_{1}+1}{2}}, & \text{if}\;x\leq \mu \text{,}\\ \frac{1-\alpha }{1-\alpha^{*}}K\left(\nu_{2}\right){\left\{1+\frac{1}{\nu_{2}}{\left[\frac{x-\mu }{2\sigma \left(1-\alpha^{*}\right)}\right]}^{2}\right\}}^{-\frac{\nu_{2}+1}{2}}, & \text{if}\;x>\mu \end{array}$$
  for −∞ < *x* < ∞, −∞ < *μ* < ∞, 0 < *α* < 1, *ν*_1_ > 0 and *ν*_2_ > 0, where
  $$\alpha^{*}=\frac{\alpha K(\nu_{1})}{\alpha K(\nu_{1})+\left(1-\alpha \right)K(\nu_{2})};$$
- the normal inverse gamma distribution [51] with
  $$f\left(x\right)=\frac{{\left(\gamma /\delta \right)}^{\lambda }\;\alpha }{\sqrt{2\pi }K_{-1/2}\left(\delta \gamma \right)}{\left[\delta^{2}+{\left(x-\mu \right)}^{2}\right]}^{-1}\;K_{-1}\left(\alpha \sqrt{\delta^{2}+{\left(x-\mu \right)}^{2}}\right)$$
  for −∞ < *x* < ∞, −∞ < *μ* < ∞, *δ* > 0, *α* > 0 and *β* > 0, where $\gamma =\sqrt{\alpha^{2}-\beta^{2}}$ and *K*_*ν*_(⋅) denotes the modified Bessel function of the second kind of order *ν* defined by
  $$K_{\nu }\left(x\right)=\{\begin{array}{cc} \frac{\pi \text{csc}(\pi \nu )}{2}[I_{-\nu }\left(x\right)-I_{\nu }\left(x\right)], & \text{if}\;\nu \;\notin \;Z\text{,}\\ \lim_{\mu \to \nu }K_{\mu }\left(x\right), & \text{if}\;\nu \;\in \;Z\text{,} \end{array}$$
  where *I*_*ν*_(⋅) denotes the modified Bessel function of the first kind of order *ν* defined by
  $$I_{\nu }\left(x\right)=\overset{\infty }{\underset{k=0}{\sum }}\frac{1}{\Gamma \left(k+\nu +1\right)k!}{\left(\frac{x}{2}\right)}^{2k+\nu };$$
- the hyperbolic distribution [51] with
  $$f\left(x\right)=\frac{{\left(\gamma /\delta \right)}^{\lambda }\;\alpha^{-1/2}}{\sqrt{2\pi }K_{1}\left(\delta \gamma \right)}{\left[\delta^{2}+{\left(x-\mu \right)}^{2}\right]}^{1/2}\;K_{1/2}\left(\alpha \sqrt{\delta^{2}+{\left(x-\mu \right)}^{2}}\right)$$
  for −∞ < *x* < ∞, −∞ < *μ* < ∞, *δ* > 0, *α* > 0 and *β* > 0, where $\gamma =\sqrt{\alpha^{2}-\beta^{2}}$;
- the generalized hyperbolic distribution [51] with
  $$f\left(x\right)=\frac{{\left(\gamma /\delta \right)}^{\lambda }\;\alpha^{1/2-\lambda }}{\sqrt{2\pi }K_{\lambda }\left(\delta \gamma \right)}{\left[\delta^{2}+{\left(x-\mu \right)}^{2}\right]}^{\lambda -1/2}\;K_{\lambda -1/2}\left(\alpha \sqrt{\delta^{2}+{\left(x-\mu \right)}^{2}}\right)$$
  for −∞ < *x* < ∞, −∞ < *μ* < ∞, −∞ < λ < ∞, *δ* > 0, *α* > 0 and *β* > 0, where $\gamma =\sqrt{\alpha^{2}-\beta^{2}}$.

Several of these distributions are nested: the normal distribution is the limiting case of the Student’s *t* distribution as *ν* → ∞; the normal distribution is the particular case of the exponential power distribution for *β* = 2; the Laplace distribution is the particular case of the exponential power distribution for *β* = 1; the normal distribution is the particular case of the skew normal distribution for λ = 0; the Student’s *t* distribution is the particular case of the skew *t* distribution for λ = 0; the exponential power distribution is the particular case of the skewed exponential power distribution for *α* = 1/2; the Student’s *t* distribution is the particular case of the generalized *t* distribution for *τ* = 2; the skewed exponential power distribution is the particular case of the asymmetric exponential power distribution for *p*_1_ = *p*_2_; the Student’s *t* distribution is the particular case of the skewed Student’s *t* distribution for *α* = 1/2; the skewed Student’s *t* distribution is the particular case of the asymmetric Student’s *t* distribution for *ν*_1_ = *ν*_2_; the skewed exponential power distribution is the particular case of the asymmetric exponential power distribution for *p*_1_ = *p*_2_; the normal inverse gamma distribution is the particular case of the generalized hyperbolic distribution for λ = −1/2; the hyperbolic distribution is the particular case of the generalized hyperbolic distribution for λ = 1; and so on.

The fifteen distributions include heavy tailed and light tailed distributions. The normal, logistic, Laplace, exponential power, skew normal, skewed exponential power and asymmetric exponential power distributions have light tails. The Student’s *t*, skew *t*, generalized *t*, skewed Student’s *t*, asymmetric Student’s *t*, normal inverse gamma, hyperbolic and generalized hyperbolic distributions have heavy tails.

Each distribution was fitted by the method of maximum likelihood. That is, if *x*_1_, *x*_2_, …, *x*_*n*_ are independent observations on *X* then the parameters of each distribution are the values maximizing the likelihood

$$L\;(\Theta )=\prod_{i=1}^{n}f(x_{i};\Theta )$$

or the log-likelihood

$$\ln L\;(\Theta )=\sum_{i=1}^{n}\ln f\;(x_{i};\Theta ),$$

where $\Theta =\left(\theta_{1},\theta_{2},\ldots ,\theta_{k}\right)\prime$ is a vector of parameters specifying *f*(⋅). We shall let $\hat{\Theta }=\left(\hat{\theta_{1}},\hat{\theta_{2}},\ldots ,\hat{\theta_{k}}\right)\prime$ denote the maximum likelihood estimate of **Θ**. The maximization was performed using the routine nlm in the R software package [52]. The standard errors of $\hat{\Theta }$ were computed by approximating the covariance matrix of $\hat{\Theta }$ by the inverse of observed information matrix, i.e.,

$$\text{cov}\;\left(\hat{\Theta }\right)\approx {{\left(\begin{array}{cccc} \frac{\partial^{2}\text{ln}L}{\partial \theta_{1}^{2}} & \frac{\partial^{2}\text{ln}L}{\partial \theta_{1}\partial \theta_{2}} & \cdots & \frac{\partial^{2}\text{ln}L}{\partial \theta_{1}\partial \theta_{k}}\\ \frac{\partial^{2}\text{ln}L}{\partial \theta_{2}\partial \theta_{1}} & \frac{\partial^{2}\text{ln}L}{\partial \theta_{2}^{2}} & \cdots & \frac{\partial^{2}\text{ln}L}{\partial \theta_{2}\partial \theta_{k}}\\ \vdots & \vdots & \ddots & \vdots \\ \frac{\partial^{2}\text{ln}L}{\partial \theta_{k}\partial \theta_{1}} & \frac{\partial^{2}\text{ln}L}{\partial \theta_{k}\partial \theta_{2}} & \cdots & \frac{\partial^{2}\text{ln}L}{\partial \theta_{k}^{2}} \end{array}\right)}^{-1}|}_{{}_{\Theta =\hat{\Theta }}}.$$

Many of the fitted distributions are not nested. Discrimination among them was performed using various criteria:

- the Akaike information criterion due to [53] defined by
  $$\text{AIC}=2k-2\ln L\;(\hat{\Theta });$$
- the Bayesian information criterion due to [54] defined by
  $$\text{BIC}=k\ln n-2\ln L\;(\hat{\Theta });$$
- the consistent Akaike information criterion (CAIC) due to [55] defined by
  $$\text{CAIC}=-2\ln L\;(\hat{\Theta })+k\;(\ln n+1);$$
- the corrected Akaike information criterion (AICc) [56] defined by
  $$\text{AICc}=\text{AIC}+\frac{2k(k+1)}{n-k-1};$$
- the Hannan-Quinn criterion [57] defined by
  $$\text{HQC}=-2\ln L\;(\hat{\Theta })+2k\ln\ln n;$$
- the Kolmogorov-Smirnov statistic [58], [59] defined by
  $$\text{KS}=\underset{x}{\sup}|\frac{1}{n}\sum_{i=1}^{n}I\{x_{i}\leq x\}-\hat{F}\left(x\right)|,$$
  where *I*{⋅} denotes the indicator function and $\hat{F}(\cdot )$ the maximum likelihood estimate of *F*(*x*);
- the Anderson-Darling statistic [60] defined by
  $$\text{AD}=-n-\sum_{i=1}^{n}\{\ln\hat{F}(x_{\left(i\right)})+\ln[1-\hat{F}(x_{\left(n+1-i\right)})]\},$$
  where *x*_(1)_ ≤ *x*_(2)_ ≤ ⋯ ≤ *x*_(*n*)_ are the observed data arranged in increasing order.

The smaller the values of these criteria the better the fit. For more discussion on these criteria, see [61] and [62].

The likelihood ratio test [63] can be used to discriminate among nested distributions. According to this test, if Distribution 1 has *k*_1_ parameters and yields a log-likelihood of ln *L*_1_ and Distribution 2, a particular case of Distributions 1, has *k*_2_ < *k*_1_ parameters and yields a log-likelihood of ln *L*_2_, then the former should be preferred if $2\left(\text{ln}L_{1}-\text{ln}L_{2}\right)>\chi_{{}^{{}_{k_{1}-k_{2},0.95}}}^{2}$, where $\chi_{{}^{{}_{\nu ,\alpha }}}^{2}$ denotes the 100*α* percentile of a chi-square random variable with *ν* degrees of freedom.

## Results and Discussion

The fifteen distributions in Section 3 were fitted to the data described in Section 2. The method of maximum likelihood was used. The log-likelihood values, the values of AIC, AICc, BIC, HQC, CAIC and the *p*-values of KS, AD for the fitted distributions are shown in Table 5. The parameter estimates and their standard errors for the fitted distributions are shown in Table 6.

**Table 5:** Log-likelihoods and the five criteria for the fitted distributions.

Distribution	−ln *L*	AIC	AICc	BIC	HQC	CAIC	KS	AD
Normal	1196.425	2396.851	2396.863	2406.564	2400.551	2408.564	0.009	0.011
Student *t*	-1554.827	-3103.653	-3103.628	-3089.084	-3098.102	-3086.084	0.138	0.134
Logistic	-1391.531	-2779.063	-2779.05	-2769.35	-2775.362	-2767.35	0.024	0.036
Laplace	-1497.184	-2990.368	-2990.355	-2980.655	-2986.667	-2978.655	0.119	0.080
EP	-1560.005	-3114.01	-3113.985	-3099.441	-3108.459	-3096.441	0.218	0.325
Skew normal	-1196.425	-2386.851	-2386.825	-2372.281	-2381.299	-2369.281	0.019	0.018
Skew *t*	-1556.337	-3104.674	-3104.632	-3085.249	-3097.273	-3081.249	0.177	0.149
Generalized *t*	-1565.963	-3123.926	-3123.884	-3104.5	-3116.525	-3100.5	0.378	0.406
SEP	-1560.14	-3112.28	-3112.237	-3092.854	-3104.878	-3088.854	0.216	0.276
AEP	-1567.824	-3125.648	-3125.584	-3101.365	-3116.396	-3096.365	0.472	0.418
SST	-1556.808	-3105.616	-3105.573	-3086.19	-3098.214	-3082.19	0.199	0.205
AST	-1558.258	-3106.516	-3106.452	-3082.233	-3097.264	-3077.233	0.211	0.268
NIG	-1565.278	-3122.557	-3122.514	-3103.131	-3115.155	-3099.131	0.334	0.341
Hyperbolic	-1497.392	-2986.783	-2986.741	-2967.357	-2979.381	-2963.357	0.106	0.073
Generalized hyperbolic	-1570.229	-3130.458	-3130.395	-3106.176	-3121.206	-3101.176	0.484	0.483

**Table 6:** Fitted distributions, parameter estimates and standard errors.

Distribution	Parameter estimates and standard errors
Normal	$\hat{\mu }=4.534\times 10^{-3}\left(2.228\times 10^{-3}\right)$, $\hat{\sigma }=6.868\times 10^{-2}\left(1.581\times 10^{-3}\right)$
Student *t*	$\hat{\nu }=1.389\left(1.026\times 10^{-1}\right)$, $\hat{\mu }=3.858\times 10^{-3}\left(9.195\times 10^{-4}\right)$, $\hat{\sigma }=2.134\times 10^{-2}\left(1.197\times 10^{-3}\right)$
Logistic	$\hat{\mu }=5.391\times 10^{-3}\left(1.540\times 10^{-3}\right)$, $\hat{\sigma }=2.892\times 10^{-2}\left(8.345\times 10^{-4}\right)$
Laplace	$\hat{\mu }=3.753\times 10^{-3}\left(1.170\times 10^{-3}\right)$, $\hat{\sigma }=3.804\times 10^{-2}\left(1.241\times 10^{-3}\right)$
EP	$\hat{\mu }=3.996\times 10^{-3}\left(1.490\times 10^{-4}\right)$, $\hat{\sigma }=2.819\times 10^{-2}\left(1.368\times 10^{-3}\right)$, $\hat{\beta }=5.871\times 10^{-1}\left(2.982\times 10^{-2}\right)$
Skew normal	$\hat{\mu }=4.534\times 10^{-3}\left(3.256\times 10^{-1}\right)$, $\hat{\sigma }=6.868\times 10^{-2}\left(1.597\times 10^{-3}\right)$, $\hat{\lambda }=6.006\times 10^{-9}\left(5.942\right)$
Skew *t*	$\hat{\mu }=9.774\times 10^{-4}\left(1.865\times 10^{-3}\right)$, $\hat{\sigma }=2.133\times 10^{-2}\left(1.206\times 10^{-3}\right)$, $\hat{\lambda }=1.639\times 10^{-1}\left(9.492\times 10^{-2}\right)$, $\hat{\nu }=1.379\left(1.015\times 10^{-1}\right)$
Generalized *t*	$\hat{\mu }=3.026\times 10^{-3}\left(1.186\times 10^{-3}\right)$, $\hat{\sigma }=2.310\times 10^{-2}\left(3.695\times 10^{-3}\right)$, $\hat{\tau }=9.471\times 10^{-1}\left(1.541\times 10^{-1}\right)$, $\hat{\nu }=3.042\left(1.423\right)$
SEP	$\hat{\mu }=4.000\times 10^{-3}\left(1.507\times 10^{-4}\right)$, $\hat{\sigma }=2.812\times 10^{-2}\left(1.366\times 10^{-3}\right)$, $\hat{p}=5.842\times 10^{-1}\left(2.963\times 10^{-2}\right)$, $\hat{\alpha }=4.936\times 10^{-1}\left(1.298\times 10^{-2}\right)$
AEP	$\hat{\mu }=9.067\times 10^{-4}\left(3.054\times 10^{-4}\right)$, $\hat{\sigma }=2.776\times 10^{-2}\left(1.363\times 10^{-3}\right)$, $\hat{p_{1}}=5.435\times 10^{-1}\left(2.813\times 10^{-2}\right)$, $\hat{p_{2}}=6.028\times 10^{-1}\left(3.706\times 10^{-2}\right)$, $\hat{\alpha }=4.462\times 10^{-1}\left(1.464\times 10^{-2}\right)$
SST	$\hat{\mu }=1.945\times 10^{-3}\left(1.299\times 10^{-3}\right)$, $\hat{\sigma }=6.295\times 10^{-2}\left(3.105\times 10^{-3}\right)$, $\hat{\nu }=1.380\left(1.015\times 10^{-1}\right)$, $\hat{\alpha }=4.627\times 10^{-1}\left(1.859\times 10^{-2}\right)$
AST	$\hat{\mu }=2.394\times 10^{-4}\left(1.557\times 10^{-3}\right)$, $\hat{\sigma }=6.310\times 10^{-2}\left(3.112\times 10^{-3}\right)$, $\hat{\nu_{1}}=1.193\left(1.331\times 10^{-1}\right)$, $\hat{\nu_{2}}=1.593\left(1.804\times 10^{-1}\right)$, $\hat{\alpha }=4.363\times 10^{-1}\left(2.329\times 10^{-2}\right)$
NIG	$\hat{\mu }=3.504\times 10^{-3}\left(2.305\times 10^{-4}\right)$, $\hat{\delta }=2.070\times 10^{-2}\left(1.055\times 10^{-3}\right)$, $\hat{\alpha }=3.916(2.989)$, $\hat{\beta }=1.976\times 10^{-1}\left(1.003\times 10^{-1}\right)$
Hyperbolic	$\hat{\mu }=3.023\times 10^{-3}\left(6.032\times 10^{-4}\right)$, $\hat{\delta }=1.068\times 10^{-5}\left(9.945\times 10^{-3}\right)$, $\hat{\alpha }=2.628\times 10^{1}\left(1.207\times 10^{1}\right)$, $\hat{\beta }=5.185\times 10^{-1}\left(3.492\times 10^{-1}\right)$
Generalized hyperbolic	$\hat{\mu }=2.948\times 10^{-3}\left(8.964\times 10^{-4}\right)$, $\hat{\delta }=1.217\times 10^{-2}\left(2.578\times 10^{-3}\right)$, $\hat{\alpha }=7.731(1.517)$, $\hat{\beta }=3.447\times 10^{-1}\left(5.186\times 10^{-1}\right)$, $\hat{\lambda }=-1.390\times 10^{-1}\left(1.112\times 10^{-1}\right)$

We can see from Table 5 that the Laplace distribution gives the smallest values for −ln *L*, AIC, AICc, BIC, HQC, CAIC and the largest *p*-values among all the two-parameter distributions. The exponential power distribution gives the smallest values for −ln *L*, AIC, AICc, BIC, HQC, CAIC and the largest *p*-values among all the three-parameter distributions. The generalized *t* distribution gives the smallest values for −ln *L*, AIC, AICc, BIC, HQC, CAIC and the largest *p*-values among all the four-parameter distributions. The generalized hyperbolic distribution gives the smallest values for −ln *L*, AIC, AICc, BIC, HQC, CAIC and the largest *p*-values among all the five-parameter distributions.

Overall, the generalized hyperbolic distribution gives the best fit by having the smallest values for −ln *L*, AIC, AICc, BIC, HQC, CAIC and the largest *p*-values. The normal distribution gives the worst fit by having the largest values for −ln *L*, AIC, AICc, BIC, HQC, CAIC and the smallest *p*-values. The skew normal distribution gives the second worst fit by having the second largest values for −ln *L*, AIC, AICc, BIC, HQC, CAIC and the second smallest *p*-values. With respect to *p*-values, all but the normal, skew normal and logistic distributions provide adequate fits at the five percent level.

The normal inverse gamma and the hyperbolic distributions are particular cases of the generalized hyperbolic distribution. The use of the likelihood ratio test shows that neither of them provide as good a fit as the generalized hyperbolic distribution.

One should not conclude that the generalized hyperbolic distribution gives the best fit because it has the largest number of parameters. Each of the five criteria (AIC, AICc, BIC, HQC, CAIC) has a factor penalizing for every new parameter added. The factor is 2 for the AIC, ln *n* for the BIC, ln *n* for the CAIC and 2 ln ln *n* for the HQC. So, more parameters do not necessarily imply better fits. The generalized hyperbolic distribution gives the best fit only because it captures the data significantly better than other distributions.

**Fig 6:**
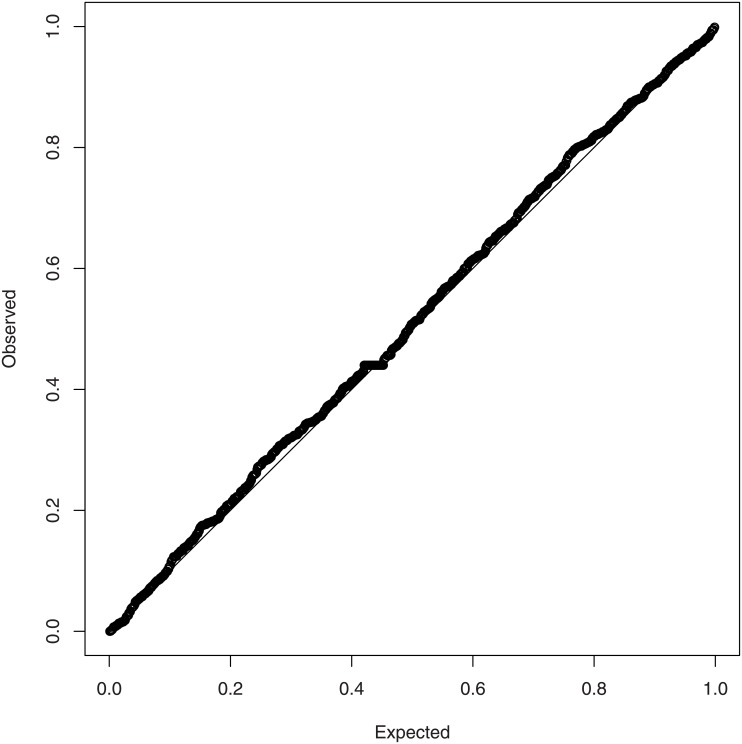
Probability plot for the fit of the generalized hyperbolic distribution.

**Fig 7:**
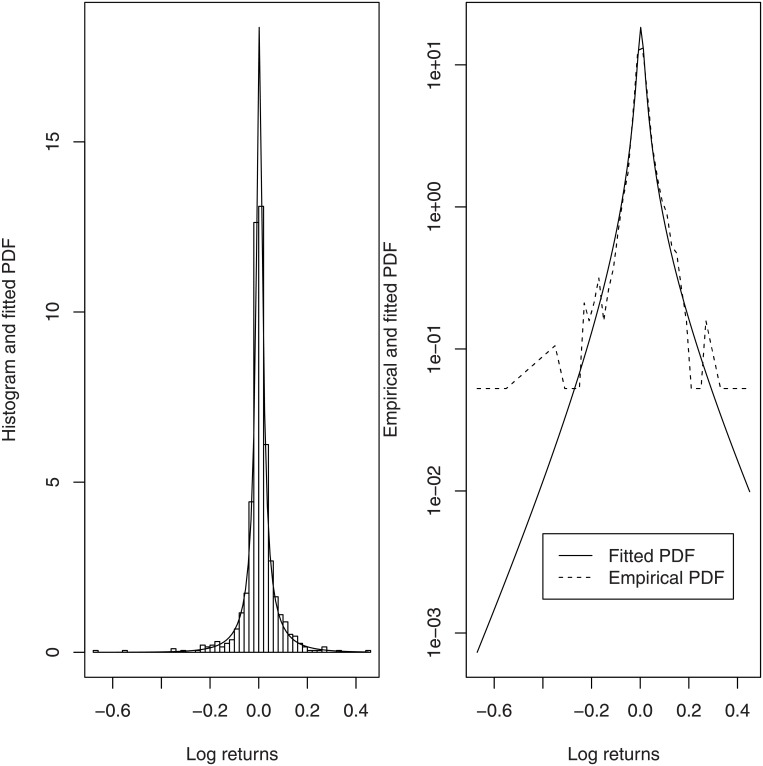
Empirical histogram and fitted pdf of the generalized hyperbolic distribution (left), Empirical pdf and fitted pdf of the generalized hyperbolic distribution plotted on log scale (right).

The probability plot and the density plot of the fitted generalized hyperbolic distribution are shown in Figs 6 and 7. The fitted pdf is also plotted on the log scale. Both figures suggest that the fit is good. The fit appears reasonable also in the tails.

**Fig 8:**
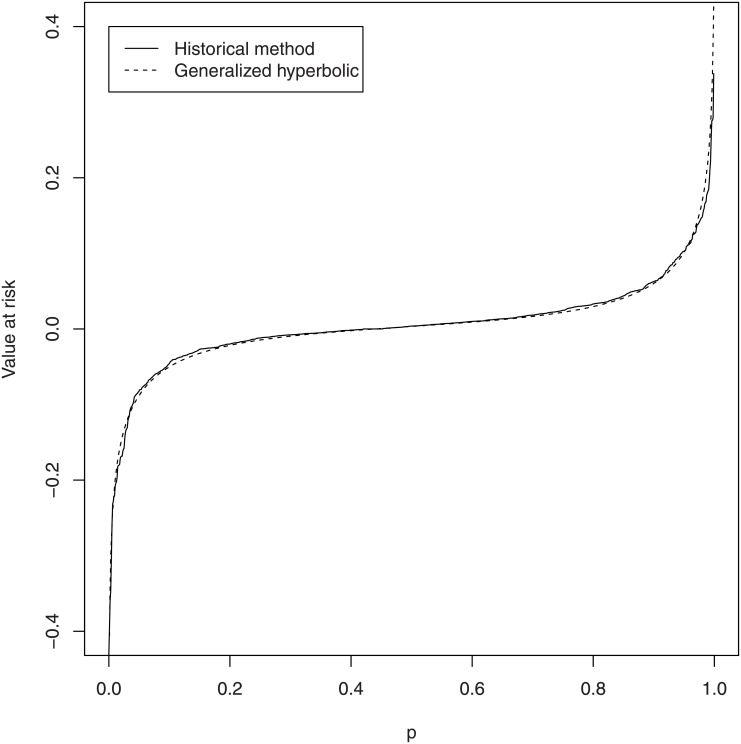
Historical estimates of the VaR and estimates based on the fitted generalized hyperbolic distribution.

**Fig 9:**
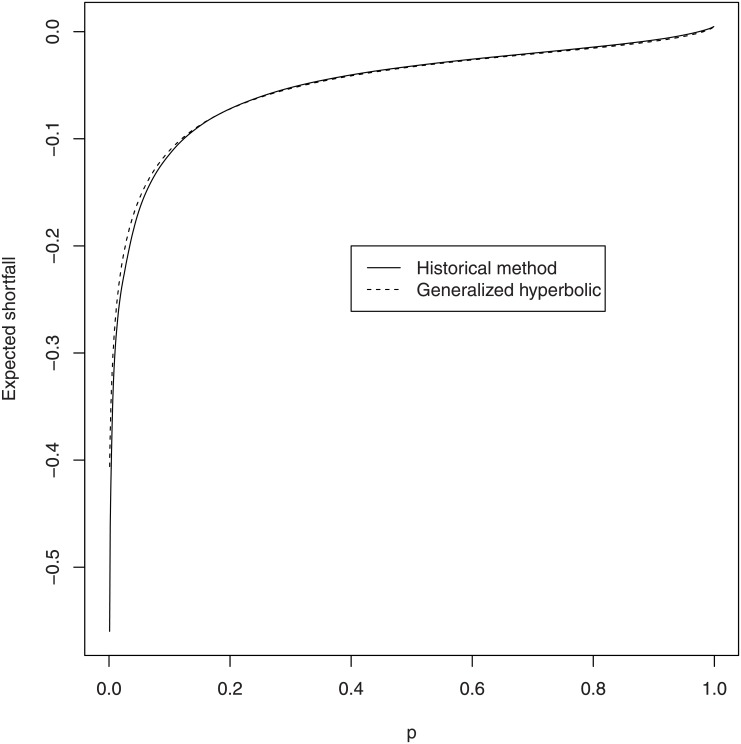
Historical estimates of the ES and estimates based on the fitted generalized hyperbolic distribution.

The value at risk (VaR) and expected shortfall (ES) are two of the most important measures of financial risk. For the best fitting distribution, the VaR and the ES with probability *p* can be estimated by

$$\frac{{\left(\hat{\gamma }/\hat{\delta }\right)}^{\hat{\lambda }}{\hat{\alpha }}^{1/2-\hat{\lambda }}}{\sqrt{2\pi }K_{\hat{\lambda }}\left(\hat{\delta }\hat{\gamma }\right)}\int_{-\infty }^{{\hat{VaR}}_{p}}\;{\left[{\hat{\delta }}^{2}+{\left(x-\hat{\mu }\right)}^{2}\right]}^{\hat{\lambda }-1/2}K_{\hat{\lambda }-1/2}\;\left(\hat{\alpha }\sqrt{{\hat{\delta }}^{2}+{\left(x-\hat{\mu }\right)}^{2}}\right)\;dx=p$$

and

$${\hat{\text{ES}}}_{p}=\frac{1}{p}\int_{0}^{p}{\hat{\text{VaR}}}_{q}dq,$$

respectively, where $\hat{\gamma }=\;\sqrt{{\hat{\alpha }}^{2}-{\hat{\beta }}^{2}}$. The plot of ${\hat{\text{VaR}}}_{p}$ versus *p* is shown in Fig 8. The plot of ${\hat{\text{ES}}}_{p}$ versus *p* is shown in Fig 9. Also shown in these figures are historical estimates of the VaR and the ES. The fitted values for the VaR and the ES appear very close to the historical estimates.

The estimates of the VaR and the ES can be inferred from Figs 8 and 9, respectively. The estimates for *p* = 0.1, 0.01, 0.001, 0.0001, 0.00001, 0.9, 0.99, 0.999, 0.9999, 0.99999 are given in Table 7. The two tails in Fig 8 are highly steeped, confirming that the returns of Bitcoin are highly volatile.

**Table 7:** Fitted estimates of VaR and ES.

*p*	VaR	ES
0.1	−5.015 × 10^−2^	−1.138 × 10^−1^
0.01	−2.043 × 10^−1^	−2.926 × 10^−1^
0.001	−4.108 × 10^−1^	−5.105 × 10^−1^
0.0001	−6.420 × 10^−1^	−7.477 × 10^−1^
0.00001	−8.865 × 10^−1^	−9.960 × 10^−1^
0.9	6.023 × 10^−2^	−9.360 × 10^−3^
0.99 range	2.282 × 10^−1^	3.432 × 10^−3^
0.999	4.539 × 10^−1^	3.975 × 10^−3^
0.9999	7.065 × 10^−1^	4.446 × 10^−3^
0.99999	9.739 × 10^−1^	4.525 × 10^−3^

An out of sample performance of these risk measures can be assessed by a backtest measure due to [64]:

$$\begin{array}{c} \frac{1}{2\tau_{\alpha }}|\sum_{x_{i}\in z_{\alpha }}[x_{i}-{\hat{\text{ES}}}_{\alpha }]|+\frac{1}{2\delta_{\alpha }}|\sum_{x_{i}\in \zeta_{\alpha }}[x_{i}-{\hat{\text{ES}}}_{\alpha }]|, \end{array}$$ (1)

where

$$\delta_{\alpha }=\sum_{i=1}^{n}\;I\;\{x_{i}-{\hat{\text{ES}}}_{\alpha }<q_{\alpha }\}$$

for *α* small,

$$\delta_{\alpha }=\sum_{i=1}^{n}\;I\;\{x_{i}-{\hat{\text{ES}}}_{\alpha }>q_{\alpha }\}$$

for *α* larger,

$$\zeta_{\alpha }=\{x_{i}:x_{i}-{\hat{\text{ES}}}_{\alpha }<q_{\alpha }\}$$

for *α* small,

$$\zeta_{\alpha }=\{x_{i}:x_{i}-{\hat{\text{ES}}}_{\alpha }>q_{\alpha }\}$$

for *α* large,

$$z_{\alpha }=\{x_{i}:x_{i}<{\hat{\text{VaR}}}_{\alpha }\}$$

for *α* small,

$$z_{\alpha }=\{x_{i}:x_{i}>{\hat{\text{VaR}}}_{\alpha }\}$$

for *α* large, and *q*_*α*_ is the *α*th empirical quantile of $\left\{x_{i}-{\hat{\text{ES}}}_{\alpha },i=1,2,\ldots ,n\right\}$. Smaller backtest measures correspond to better forecasting.

The values of Eq (1) for *α* = 0.001, 0.999 and the fifteen distributions are shown in Table 8. We see that the generalized hyperbolic distribution gives the smallest values. These values appear reasonably small. The largest values are given by the normal distribution.

**Table 8:** The backtest measure for the fitted distributions.

Distribution	*α* = 0.001	*α* = 0.999
Normal	5.908	5.335
Student *t*	3.253	3.599
Logistic	5.067	4.608
Laplace	3.819	4.063
EP	1.109	2.364
Skew normal	5.235	5.283
Skew *t*	2.979	2.921
Generalized *t*	0.589	1.686
SEP	1.739	2.624
AEP	0.093	1.492
SST	1.899	2.845
AST	1.884	2.786
NIG	0.925	2.236
Hyperbolic	4.715	4.208
Generalized hyperbolic	0.052	0.134

Finally, we give predictions for the exchange rate of Bitcoin. Let *Y*_*i*_ denote the exchange rate on the *i*th day counting from the 13th of September 2011. Then *X*_*i*_ = ln *Y*_*i*_ − ln *Y*_*i*−1_ is the log-return on the *i*th day. We can write the exchange rate on the *n*th day (counting from the 13th of September 2011) as

$$Y_{n}-Y_{0}=\exp\;(\sum_{i=1}^{n}X_{i}).$$

We suppose *Y*_0_ is a deterministic variable taking the value 5.97, the value suggested by the data set. So,

$$Y_{n}=5.97+\exp\;(\sum_{i=1}^{n}X_{i})=5.97+\exp\left(T\right)$$

say.

According to our results, *X*_*i*_ can be assumed to be independent and identical generalized hyperbolic random variables. The generalized hyperbolic random variable does have a closed form characteristic function [51]. Hence, by the inversion theorem of [65], the cdf of *T* can be expressed as

$$F_{T}\left(t\right)=\frac{1}{2}-\frac{{\hat{\gamma }}^{n\hat{\lambda }}}{\pi K_{\hat{\lambda }}^{n}\left(\hat{\delta }\hat{\gamma }\right)}\int_{0}^{\infty }s^{-1}\;\text{Im}\left\{\frac{K_{\hat{\lambda }}^{n}\left(\hat{\delta }\sqrt{{\hat{\alpha }}^{2}-{\left(\hat{\beta }+\text{i}s\right)}^{2}}\right)\;\text{exp}\left[\text{i}s\left(n\mu -t\right)\right]}{{\left[{\hat{\alpha }}^{2}-{\left(\hat{\beta }+\text{i}s\right)}^{2}\right]}^{-n\hat{\lambda }/2}}\right\}ds,$$

where $\text{i}=\sqrt{-1}$ and Im(⋅) denotes the imaginary part. The cdf of *Y*_*n*_ is therefore

$$F_{Y_{n}}\left(y\right)=F_{T}(\ln(y-5.97)).$$

Various high and low percentiles of *Y*_*n*_ for *n* = 1000, 2000, 3000, 4000, 5000 are given in Table 9. These predictions can be reliable and accurate at least in the short term given the goodness of fit to the data and given the small values of the backtest measure. The extreme worst case scenario could occur if rules and regulations prohibit Bitcoin being used or entered into countries and markets. This will lead to a deterioration in confidence of Bitcoin investors. The extreme best case scenario could lead to Bitcoin being used as an alternative for Paypal or even as the main currency in many countries.

**Table 9:** Predictions for the exchange rate of Bitcoin at day *n* (counting from the 13th of September 2011).

Percentile level	*n* = 1000	*n* = 2000	*n* = 3000	*n* = 4000	*n* = 5000
0.1	11.904	182.635	6863.276	305360	14844735
0.01	6.599	13.360	146.519	3435.949	98169.76
0.001	6.092	6.696	14.163	134.777	2508.388
0.0001	6.002	6.077	6.760	14.613	128.040
0.00001	5.980	5.990	6.074	6.798	14.838
0.9	1467.006	425537.8	95054327	18508171925	3.301 × 10^12^
0.99	13791.29	10172920	4637660718	1.648 × 10^12^	4.992 × 10^14^
0.999	71142.51	103590788	79559056488	4.388 × 10^13^	1.958 × 10^16^
0.9999	274621.4	699752429	8.256 × 10^11^	6.539 × 10^14^	4.014 × 10^17^
0.99999	887173.1	3674309279	6.293 × 10^12^	6.824 × 10^15^	5.526 × 10^18^

The numbers in Table 9 are consistent with the observations “… the most notable aspect of this forecast is the uncertainty. Confidence intervals are very wide, so the overall confidence in the point forecast is low. With such high volatility, the best an investor or user of Bitcoins could hope for is to have advance warning of dramatic crashes” of [66].

## Conclusions

We have analyzed the exchange rate of Bitcoin versus USD using fifteen of the most popular parametric distributions in finance, the most comprehensive collection of distributions ever fitted to any exchange rate data. We have found that the generalized hyperbolic distribution gives the best fit, as assessed by the log-likelihood value, AIC value, AICc value, BIC value, HQC value, CAIC value, probability plot and the density plot.

We have given predictions for the log-returns of the exchange rate based on the VaR and the ES, the two most popular financial risk measures. In particular, the log-returns will be greater than 2.282 × 10^−1^ with 1 percent chance and will be less than −2.043 × 10^−1^ with 1 percent chance. Also, the log-returns will be greater than 4.539 × 10^−1^ with 0.1 percent chance and will be less than −4.108 × 10^−1^ with 0.01 percent chance.

We have also given predictions for the exchange rate at future times taken in steps of one thousand days (approximately three years). In particular, the exchange rate in about six years from the 13th of September 2011 could exceed 10172920 with 1 percent chance and could be less than 13.36 with 1 percent chance. Also, the exchange rate in about nine years from the 13th of September 2011 could exceed 4637660718 with 1 percent chance and could be less than 146.519 with 1 percent chance.

These conclusions are consistent with the following: “Bitcoin exchange rates exhibit somewhat complicated dynamics. In the past 24 months, the USD-BTC exchange rate increased more than 50-fold” [67]; “Bitcoin investment exhibits very high volatility but also very high returns” [12].

Some future work are to use nonparametric or semiparametric distributions to analyze the exchange rate data.
